# Do ethnic chinese older adults with epithelial ovarian cancer survive a poorer prognosis?

**DOI:** 10.1186/s13048-023-01177-3

**Published:** 2023-06-06

**Authors:** Wu Huang, Yiting Bao, Xukai Luo, Liangqing Yao, Lei Yuan

**Affiliations:** Department of Gynecology, Obstetrics and Gynecology Hospital of Fudan University, Shanghai, China

**Keywords:** Age, Chinese, Older, SEER, Epithelial ovarian cancer, Overall survival, Ethnic chinese

## Abstract

**Background:**

The risk of suffering epithelial ovarian cancer (EOC) for women increases with age evidently, while the prognosis of older EOC patients remain unclear. Against the backdrop of the accelerate aging process in China, this paper investigates whether the older EOC patients have a lower overall survival probability than the younger patients based on the sample of ethnic Chinese population.

**Methods:**

A total of 323 ethnic Chinese patients diagnosed as epithelial ovarian cancer were extracted from the Surveillance, Epidemiology, and End Results (SEER) database. We compared the overall survival probability between the younger group (< 70 years) and the older patients group (≥ 70 years). Survival curves were drawn using the Kaplan-Meier method, comparisons among different subgroups were evaluated using log-rank tests, and independent prognostic factors were identified by univariate and multivariate Cox regression analyses.

**Results:**

43 patients were (13.3%) in the older patients group and 280 (86.7%) in the younger group. The distribution patterns between two groups were significantly different with regard to marital status, histologic type and FIGO stage. The median overall survival (OS) was significantly longer in the younger group than the older patients group (not reached vs. median 39 months, p < 0.05). The multivariate analysis demonstrated that the age (The older vs. the younger, HR: 1.967, P = 0.007), primary tumor laterality (HR: 1.849, P = 0.009), and FIGO stage (III vs. I, HR: 3.588, P = 0.001; and IV vs. I, HR: 4.382, P = 0.001; respectively) remained as important risk factors while Histology (HGSOC vs. CCOC, HR: 0.479, P = 0.025; and LGSOC/MOC/EC vs. CCOC, HR: 0.390, P = 0.034; respectively) and the number of lymph node dissected more than 10 was a protective factor (HR: 0.397, P = 0.008). In an analysis of 104 pairs of patients matched on the basis of the propensity score, the older patients group had significantly lower overall mortality (HR = 2.561, P = 0.002).

**Conclusion:**

Ethnic Chinese Older EOC patients have a worse prognosis than the younger patients.

**Supplementary Information:**

The online version contains supplementary material available at 10.1186/s13048-023-01177-3.

## Background

In 2020, over 313,000 women were diagnosed with ovarian cancer and nearly 207,000 died from this disease worldwide [1]. The risk of suffering ovarian cancer for women increases with age evidently. Among ovarian cancer patients, only 10 to 15% of cases are diagnosed before menopause [2]. Due to the aggressiveness of ovarian cancer and the lack of specific symptoms for early detection, fewer than one-half of women survive beyond 5 years after diagnosis [3].

Surgical staging or cytoreduction followed by adjuvant chemotherapy is the standard treatment for most patients with epithelial ovarian cancer (EOC). With increasing age, more and more people are getting comorbid conditions, polypharmacy, cognitive impairment, depression, frailty, poor nutrition, and inadequate social support [4]. Due to these potential risk factors, numerous older advanced ovarian cancer patients might obtain less aggressive therapies and have poorer disease-specific survival, compared with the younger counterparts.

Previous studies have showed that age may affect the prognosis of different tumor types, such as breast cancer [5], colon cancer [6], lung cancer [7], as well as epithelial ovarian cancer. For instance, one recent study showed that the age at the time of diagnosis younger than 40 years was an independent protective prognostic factor for ovarian cancer patients both for overall survival (OS) and progression-free survival (PFS) [8]. And in the Netherlands, In comparison with the referent age group of 60 to 74 years, the younger age group showed a better survival outcome and the older age group showed a worse one [9]. Additionally, some studies have selected 65 years old as cut-off age for older group [4, 10–12], while others using 70 years old [13, 14]. The definition of the older patients and the inclusion criteria are different in each study, which might result in inconsistent conclusions regarding the prognostic value of age. Moreover, due to data restrictions, there is limited research on the survival probability of Chinese older women with ovarian cancer yet.

Both ethnic Chinese in the US and Chinese women in China have a similar genetic background, which is closely associated with the occurrence of the cancer. Ethnicity is defined by specific physical, hereditary and cultural traditions or origins, not necessarily by birthplace, place of residence, or citizenship in SEER database. Thus, we aimed to investigate the differences in prognosis between the older and younger ethnic Chinese patients diagnosed with epithelial ovarian cancer, to offer new insights into the treatment of Chinese population.

## Data and methods

### Data source

This study is a retrospective cohort study. Medical records of patients with ovarian epithelial cancer were acquired from the Surveillance, Epidemiology, and End Results (SEER) database. The cancer patients in SEER were from 18 regional registries and covered approximately 34.6% of the total United States population [15]. Patient identification, data accumulation and entry, and rigorous quality control for the SEER Program are managed by registered trained personnel [16].

### Data extraction

Data were downloaded from SEER database (Data Incidence-SEER 18 Regs Custom Data, with additional treatment fields, Nov 2018 sub 1975–2016 varying), using the SEER*Stat software, version 8.3.9 (https://seer.cancer.gov/seerstat/). Patients with epithelial ovarian cancer from 2004 to 2016 were selected by the International Classification of Diseases for Oncology, 3rd edition (ICD-O-3) morphology codes (according to 2014 WHO EOC histological types and a prior population-based study [17]) and the ethnicity “Chinese” (ethnicity code “04” in SEER) from ovarian cancer patients.

### Measurement

**Fig. 1:**
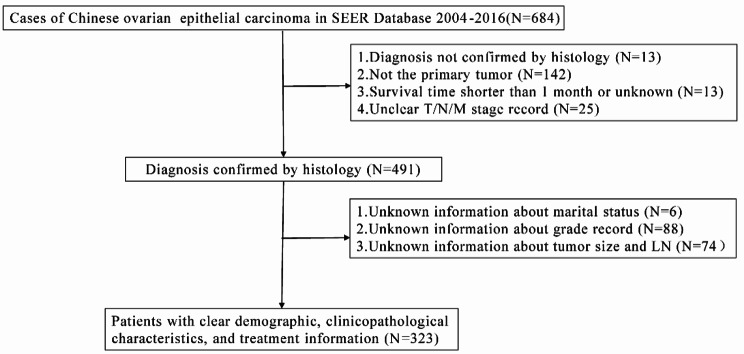
Flow chart

In this study, patients aged 70 and above at diagnosis were coded as the older patients group while those younger than age 70 were coded as the younger group. Marital status was classified into three types, the currently married, the never married and the separated/ divorced/widowed [18]. Tumor characteristics included tumor differentiation (Grade I (High differentiated), Grade II (Medium differentiated), Grade III (Low differentiated) or Grade IV (Undifferentiated)), FIGO stage (The FIGO stage was re-evaluated based on information from the database, which included TNM stage (American Joint Commission on Cancer [AJCC] 6th version and 7th version), tumor size, lymph node status and distant metastasis), ovarian tumor laterality (Unilateral versus Bilateral), performance of regional lymphadenectomy (no, adequate, and non-adequate; ≥10 excised lymph nodes was defined as adequate lymphadenectomy according to the GOG criteria [19], chemotherapy use (No/Unknown versus Yes), radiotherapy(No/Unknown versus Yes), and types of surgery (No surgery/Uncomplete surgical staging group, Complete surgical staging group, and Cytoreductive surgery group). Additionally, grade 1 tumors (well differentiated) were considered low grade and grades 2, 3, and 4 tumors were considered high grade [20]. Overall Survival (OS) was our outcome index. A total of 323 ethnic Chinese patients diagnosed with epithelial ovarian cancer aged from 18 to 92 were included in the study for further analysis (Fig. 1).

### Statistical analysis

**Fig. 2:**
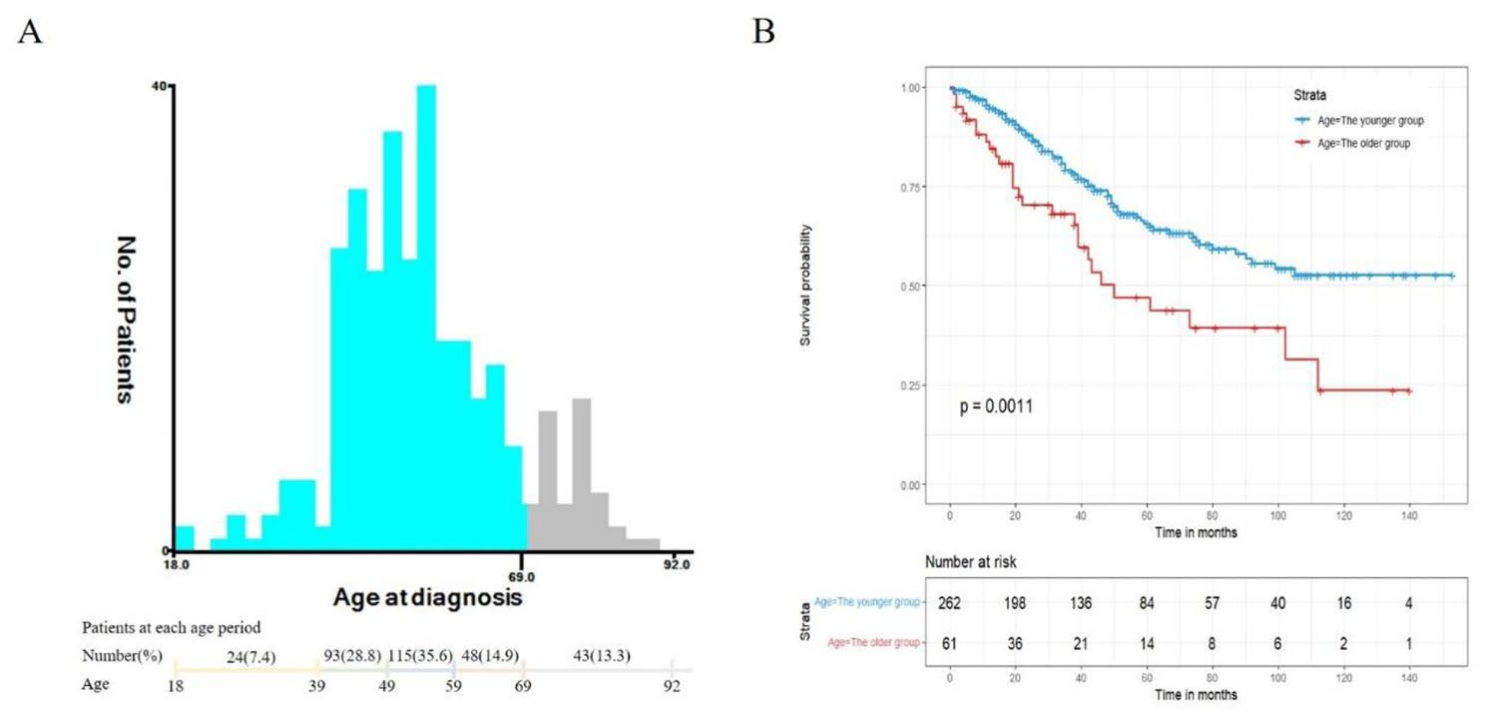
Identification of the best cut-off point of age (A) and overall survival between the younger and older (B)

The optimal cut-off point for age (18–69 years and 70–92 years) was determined by using X-tile software (version 3.6.1) [21] **(**Fig. 2**)**. Data were presented as mean ± standard deviation (SD) between groups and compared using independent-sample t tests. Two groups of categorical variables were presented as a number (n, %) and compared by chi-square (χ^2^) tests. The overall survival curve was plotted by Kaplan-Meier estimation. Additionally, univariate and multivariate Cox proportional hazard regression model were used to identify prognostic factors. We didn’t test for confounding and cox proportional hazards model with factors with a P value less than 0.1 from the univariate model was emprically used for multivariate analysis.

Additionally, we conducted a sensitivity analysis using the propensity score matching method. Using the MatchIt package, we performed a probability score matching to adjust for potential confounding. We enforced a caliper of 0.15 times the standard deviation of the propensity score to ensure closer matches. The older patients and younger patients were matched at a 1:2 ratio in propensity score matching. And the difference in overall survival between the younger and older patients groups was assessed using Cox regression model. All statistical analyses were performed with SPSS (version 24.0, SPSS, Chicago, IL, USA) and R software (version 4.0.4; http://www.r-project.org/).

## Results

### Clinicopathological characteristics and survival outcomes

We identified 323 ethnic Chinese patients diagnosed with epithelial ovarian cancer aged from 18 to 92 were included in the study, with an average age of (54.49 ± 12.31) years old and a median age of 53 years. Age in years—18–39 (24, 7.4%), 40–49 (93, 28.8%), 50–59 (115, 35.6%), 60–69 (48, 14.9%), and 70–92 (43, 13.3%) (Fig. 2A). The 5 year and 10-year survival rate are 62.5% and 49.1% respectively for all patients. Only 2 patients did not receive any surgical treatment and 23.5% of patients had ever used no/unknown chemotherapy, as shown in Table 1.

**Table 1:** Clinicopathological characteristics of the younger group and the older patients group

Variables		All patients n (%)	The younger(<70) n (%)	The older (≥ 70) n (%)	P
Total		323(100)	280(86.7)	43(13.3)	
Age (y)		54.49 ± 0.69	51.05 ± 9.08	76.86 ± 4.68	< 0.001*
Marital status					< 0.001*
	Currently married	208(64.4)	186(66.4)	22(51.2)	
	Never married	60(18.6)	58(20.7)	2(4.7)	
	SDW	36(12.9)	19(44.2)	55(17.0)	
Year of diagnosis					0.732
	2004–2009	105(32.5)	92(32.9)	13(30.2)	
	2010–2016	218(67.5)	188(67.1)	30(69.8)	
Laterality					0.363
	Unilateral	215(66.6)	189(67.5)	26(60.5)	
	Bilateral	108(33.4)	91(32.5)	17(39.5)	
Grade					0.316
	I	39(12.1)	37(13.2)	2(4.7)	
	II	93(28.8)	79(28.2)	14(32.6)	
	III	134(41.5)	113(40.4)	21(48.8)	
	IV	557(18.2)	6(14.0)	57(17.6)	
Histology					0.003*
	HGSOC	164(50.8)	131(46.8)	33(76.7)	
	LGSOC	4(1.2)	4(1.4)	0(0.0)	
	CCOC	62(19.2)	61(21.8)	1(2.3)	
	EC	64(19.8)	59(21.1)	5(11.6)	
	MOC	29(9.0)	25(8.9)	4(9.3)	
FIGO					0.001*
	Stage I	119(36.8)	113(40.4)	6(14.0)	
	Stage II	33(10.2)	28(10.0)	5(11.6)	
	Stage III	125(38.7)	106(37.9)	19(44.2)	
	Stage IV	46(14.2)	33(11.8)	13(30.2)	
Surgery					0.767
	No surgery/Uncomplete surgical staging	104(32.5)	91(32.5)	13(30.2)	
	Complete surgical staging/Cytoreductive surgery	219(67.8)	189(67.5)	30(69.8)	
LN dissected					0.232
	No or examined	87(26.9)	70(25.0)	17(39.5)	
	1–10	91(28.2)	79(28.2)	12(27.9)	
	≥ 11	145(44.9)	131(46.8)	14(32.6)	
Chemotherapy					0.964
	No/Unknow	76(23.5)	66(23.6)	10(23.3)	
	Yes	247(76.5)	214(76.4)	33(76.7)	
Radiation					0.295
	None/Unknown	316(97.8)	273(97.5)	43(100.0)	
	Beam radiation	7(2.2)	7(2.5)	0(0.0)	
Cause of death					< 0.001*
	alive	139(43.0)	139(46.8)	8(18.6)	
	dead due to cancer	171(52.9)	142(50.7)	29(67.4)	
	dead due to other	8(2.5)	3(1.1)	5(11.6)	
	missing/unknown	5(1.5)	4(1.4)	1(2.3)	

There were 43 patients (18.9%) in the older patients group, and 280 (86.7%) in the younger group. The average age of the younger group was 51.05 years old, as compared with the mean age of 76.86 for the older patients group. The proportion of those who were separated, divorced or widowed in the older patients group was significantly higher than the younger group (P < 0.001).

The most common epithelial ovarian cancer histologic type was high-grade serous ovarian cancer (HGSOC), which accounted for 76.7% among the older patients group. Even though clear cell (21.8%) and endometrioid (21.1%) tumors were more common in younger patients, the most common is still high-grade serous (46.8%). Low-grade serous ovarian cancer (LGSOC) was the most infrequent histologic type, with only 4 patients in the younger group. Mucinous ovarian cancer (MOC) accounted for 8.9% and 9.3% in the younger and older patients group, respectively. The percentage of patients with advanced FIGO stage was significantly higher in the older patients group than the younger group. (P = 0.001). Additionally, five older patients (2.5%) died due to other diseases (including cerebrovascular diseases and diseases of heart) while only 1.1% younger patients died of them (< 0.001).

It was not significantly different in the distribution of year of diagnosis, laterality, histological grading, surgery range, chemotherapy, radiotherapy, and the number of lymph dissected between these two groups.

### Comparison of overall survival between the older patients and younger patients

Kaplan-Meier curve of overall survival (OS) showed that the median survival of the older patients and younger patients were 39 months and not reached respectively. It further revealed that OS was significantly lower in the older patients group than the younger group (Fig. 2B).

### Prognostic factors for the patients

Univariate Cox regression analysis revealed that age at diagnosis (HR (95% CI) :1.029(1.017–1.044), P < 0.001) (as continuous variables) was significantly associated with OS. Additionally, age, primary tumor laterality, tumor histological grading, histology, FIGO stage, surgical range and the number of lymph node dissected were significantly associated with OS in our study Univariate Cox regression. The multivariate analysis demonstrated that age(HR: 1.967, P = 0.007), primary tumor laterality(HR: 1.849, P = 0.009), and FIGO stage (III vs. I, HR: 3.588, P = 0.001; and IV vs. I, HR: 4.382, P = 0.001;, respectively) remained as important risk factors while Histology (HGSOC vs. CCOC, HR: 0.479, P = 0.025; and LGSOC/MOC/EC vs. CCOC, HR: 0.390, P = 0.034; respectively) and the number of lymph node dissected more than 10 was a protective factor (HR: 0.397, P = 0.008). (Table 2)

**Table 2:** Univariate and multivariate Cox regression analysis for the overall survival of ethnic Chinese patients

		Univariate		Multivariable	
		HR (95% CI)	P	HR (95% CI)	P
Age					
	The younger	Reference		Reference	
	The older	3.406(2.178–5.326)	< 0.001	1.967(1.205–3.210)	0.007
Marital status					
	Currently married	Reference			
	Never married	0.722(0.413–1.264)	0.254		
	SDW	1.260(0.785–2.022)	0.339		
Year of diagnosis					
	2004–2009	Reference		Reference	
	2010–2016	1.466(0.961–2.237)	0.076	1.443(0.909–2.291)	0.119
Laterality					
	Unilateral	Reference		Reference	
	Bilateral	3.726(2.515–5.519)	< 0.001	1.849(1.164–2.938)	0.009
Grade					
	I	Reference		Reference	
	II	2.522(0.718–8.851)	0.149	1.436(0.350–5.898)	0.616
	III	5.739(1.795–18.347)	0.003	1.508(0.388–5.867)	0.553
	IV	6.277(1.929–20.423)	0.002	1.241(0.320–4.812)	0.755
Histology					
	CCOC	Reference		Reference	
	HGSOC	1.747(1.033–2.956)	0.038	0.479(0.251–0.912)	0.025
	LGSOC/MOC/EC	0.365(0.174–0.764)	0.008	0.390(0.164–0.930)	0.034
FIGO					
	I	Reference		Reference	
	II	1.972(0.749–5.190)	0.169	2.132(0.787–5.775)	0.136
	III	6.275(3.441–11.442)	< 0.001	3.588(1.705–7.550)	0.001
	IV	8.244(4.224–16.092)	< 0.001	4.382(1.877–10.227)	0.001
Surgery					
	No surgery/Uncomplete surgical staging	Reference		Reference	
	Complete surgical staging/Cytoreductive surgery	1.684(1.092–2.597)	0.018	1.579(0.934–2.670)	0.088
LN_dissected					
	No or examined	Reference		Reference	
	1–10	0.497(0.308–0.802)	0.004	0.605(0.349–1.048)	0.073
	≥ 11	0.388(0.248–0.605)	< 0.001	0.450(0.266–0.761)	0.003
Chemotherapy					
	No/Unknow	Reference			
	Yes	0.676(0.416–1.101)	0.116		
Radiation					
	None/Unknown	Reference			
	Beam radiation	0.565(0.139–2.291)	0.424		

### Sensitivity analysis by Propensity score matching

**Fig. 3:**
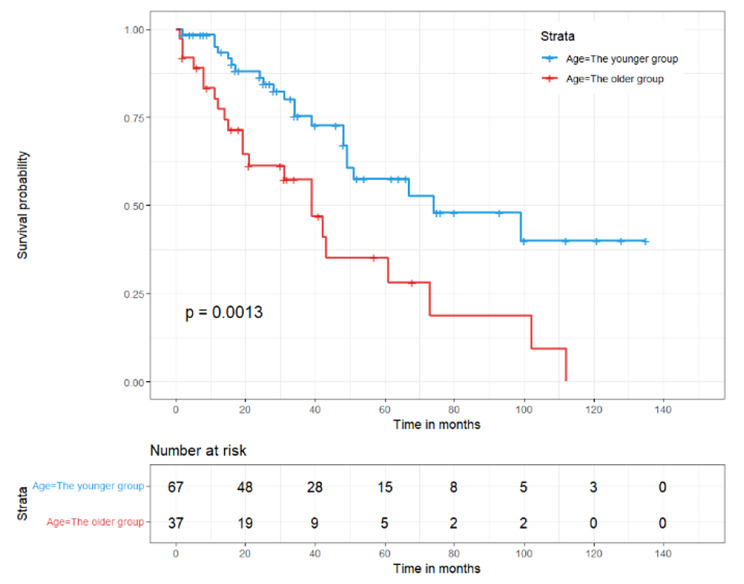
Overall survival of the propensity score-matched cohort between the younger and older

After propensity score matching for the full cohort, there were 37 older patients and 67 younger patients. In this matched cohort, the two groups did not differ significantly in terms of baseline characteristics and primary treatment modalities (Table 3). For the matched pairs, the older patients group showed significantly lower overall survival than the younger patients group (HR = 2.561, P = 0.002) (Table 4; Fig. 3).

**Table 3:** Clinicopathological characteristics of the propensity score-matched cohort

Variables		All patients n (%)	The younger(<70) n (%)	The older (≥ 70) n (%)	P
Total		104(100)	67(64.4)	37(35.6)	
Marital status					0.819
	Currently married	64 (61.5)	42 (62.7)	22 (59.5)	
	Never married	7 (6.7)	5 (7.5)	2 (5.4)	
	SDW	33 (31.7)	20 (29.9)	13 (35.1)	
Year of diagnosis					1.000
	2004–2009	30 (28.8)	19 (28.4)	11 (29.7)	
	2010–2016	74 (71.2)	48 (71.6)	26 (70.3)	
Laterality					0.833
	Unilateral	59 (56.7)	37 (55.2)	22 (59.5)	
	Bilateral	45 (43.3)	30 (44.8)	15 (40.5)	
Grade					0.933
	I	5 (4.8)	3 (4.5)	2 (5.4)	
	II	32 (30.8)	22 (32.8)	10 (27.0)	
	III	50 (48.1)	31 (46.3)	19 (51.4)	
	IV	17 (16.3)	11 (16.4)	6 (16.2)	
Histology					0.762
	HGSOC	103 (99.0)	67 (100.0)	36 (97.3)	
	LGSOC	0(0.0)	0(0.0)	0(0.0)	
	CCOC	1 (1.0)	0 (0.0)	1 (2.7)	
	EC	0(0.0)	0(0.0)	0(0.0)	
	MOC	0(0.0)	0(0.0)	0(0.0)	
FIGO					0.933
	Stage I	18 (17.3)	12 (17.9)	6 (16.2)	
	Stage II	12 (11.5)	7 (10.4)	5 (13.5)	
	Stage III	48 (46.2)	32 (47.8)	16 (43.2)	
	Stage IV	26 (25.0)	16 (23.9)	10 (27.0)	
Surgery					0.833
	No surgery/Uncomplete surgical staging	31 (29.8)	19 (28.4)	12 (32.4)	
	Complete surgical staging/Cytoreductive surgery	73 (70.2)	48 (71.6)	25 (67.6)	
LN dissected					0.728
	No or examined	36 (34.6)	22 (32.8)	14 (37.8)	
	1–10	29 (27.9)	18 (26.9)	11 (29.7)	
	≥ 11	39 (37.5)	27 (40.3)	12 (32.4)	
Chemotherapy					0.906
	No/Unknow	78 (75.0)	51 (76.1)	27 (73.0)	
	Yes	26 (25.0)	16 (23.9)	10 (27.0)	
Radiation					NA
	None/Unknown	104 (100.0)	67 (100.0)	37 (100.0)	
	Beam radiation	0(0.0)	0(0.0)	0(0.0)	

NA, not applicable

**Table 4:** Cox regression analysis of the propensity score-matched cohort

		Univariate Analysis	
		HR (95% CI)	P
Age			
	The younger	Reference	
	The older	2.561(1.411–4.649)	0.002

## Discussion

With sustained low fertility and prolonged life expectancy, the proportion of the older population has increased worldwide. Therefore, how to alleviate disease burden and promote healthy aging thus becomes a priority for aging societies. For women, gynecological malignancies account for a large proportion of all tumors, with epithelial ovarian cancer coupled with the highest mortality [22]. Previous studies have revealed that the risk of suffering from ovarian cancer might increase with age for European or American population [2]. China stands out as one of the fastest aging societies due to compressed fertility and mortality transition. However, due to data restrictions, there is limited research on the survival probability of Chinese older women with ovarian cancer yet.

Due to the unavailability of data on Chinese ovarian cancer patients, we had to resort to ethnic Chinese ovarian cancer patients in the United State, given the fact that the ethnic Chinese share the similar genetic background to Chinese women and genetic mutation is closely associated with the occurrence of the cancer. Thus, we compared the overall survival probability between the older and the younger ethnic Chinese patients diagnosed with epithelial ovarian cancer from the SEER database to offer new evidences on Chinese population.

Our study showed that EOC patients aged 70 and above had significantly worse prognosis and higher risk of death, compared with younger ones (OS: median 39 months vs. not reached). In multivariate Cox regression analysis, age, primary tumor laterality, and FIGO stage, were independent risk factors while histology and the number of lymph node dissected more than 10 was an independent protective factor.

The treatment strategy for the older ovarian cancer patients remains controversial nowadays, largely because the older patients might have more coexisting comorbid conditions and are less able to tolerate the side-effects of treatment. Therefore, the physicians might be less inclined to provide standardized treatment for older ovarian cancer therapy at the baseline. Older patients are more likely to suffer polypharmacy, cognitive impairment, depression, and to receive less social support, which in turn lead to intolerance of subsequent treatment. A recent study in the context of America also showed that older women aged ≥ 70 years old had significantly higher Cumulative Illness Rating Scale-Geriatric score, less completion of adjuvant chemotherapy, less intraperitoneal (IP) therapy, and less clinical trial participation, and thus they were less likely to have optimal cytoreductive surgery (CRS) with same surgical complexity [23].

In our study, we further compared the non-cause of death between older patients and younger patients. In the older patients group, four patients died of heart disease and one patient of cerebrovascular disease (8.2%) while the proportion in younger group was only 1.1% (one patient died of heart disease, one died of cerebrovascular disease and one died of other cause). Patients with comorbidity were more unlikely to tolerate treatment toxicity, and at the same time, treatments may also exacerbate existing comorbidity.

As a result, in view of the worse perioperative morbidity and mortality, older patients may have less opportunity to complete surgical cytoreduction than younger patients [24]. They may also receive reduced dose adjuvant chemotherapy and with chemotherapy treatment delays [25, 26]. A previous study showed that if the older patients were able to tolerate procedure, they may have similar rates of response to initial chemotherapy, platinum sensitivity, and overall survival to younger counterparts [27]. After adjustment of FIGO stage, performance status and first-line treatment received, age was no longer an independent risk factor for OS [28]. Whereas in other research, younger women with epithelial ovarian cancer have a survival advantage compared to older patients [9, 29]. Our study confirmed that older EOC patients have a worse prognosis than the younger patients.

In our study, there was no difference in the proportion of patients surgery range, using chemotherapy and performance of regional lymphadenectomy between the two groups. Patients with adequate lymphadenectomy had longer overall survival time both in the younger and older patients group. Taken together, although the role of lymphadenectomy requires further investigation, age should not be the excuse to forgo systematic lymphadenectomy or other treatments.

On the other hand, EOC consists of a heterogeneous group of neoplasms with multiple histologic subtypes [30]. The differences of tumor biology may also be associated with inherent platinum resistance [31]. It is worth noting that clear cell cancer constitutes a larger percentage of ovarian cancers in East Asia and have a poorer prognosis compared to serous cancers [19, 32]. Our study was concordant with previous ones [23, 33]. In our study, there was also difference in histological subtypes between older patients and younger groups. The older patients were more likely to be diagnosed with an advanced disease as well as more invasive and aggressive histological subtype.

The major strength of this study is the first to analyze the difference in prognosis between the younger and the older ethnic Chinese patients with EOC. In addition, our research provides evidence supporting the optimal cut-off point age for younger and older ovarian cancer patients group with 70 years old as a threshold. Furthermore, statistical analysis was performed using a double-robust adjustment with covariate adjustment and propensity score matching.

There are several limitations in this study, therefore our findings deserve more cautious interpretations. First, SEER data is retrospective, and the SEER database was not designed for our specific purpose, which could introduce inherent biases [34]. For example, patient’s comorbidities or other cofounding factors, such as performance status was not included in our study. Additionally, the data of surgery, radiation therapy, and chemotherapy in the database only had the results of YES and NO or Unknown and lacked detailed treatment plans. Moreover, as there is no data of Chinese patients with ovarian cancer available, this study used the sample of ethnic Chinese patients instead to shed light on the association between age and cancer prognosis. However, due to differences in economic and medical services between China and the U.S. Our findings among the ethnic Chinese patients may not be fully generalized to Chinese patients with ovarian cancer. Another limitation of our study is that in our real data analyses, no independent cohorts are available to do validation. We hope to validate our cutoff in the future in larger cohorts.

## Conclusion

In conclusion, older EOC patients have a significantly lower overall survival probability than the younger patients among the ethnic Chinese population. Because older patients are more likely to develop aggressive histological subtype and progress to an advanced stage. Extrapolation of these results to Chinese populations remains uncertain. Further studies are needed to investigate the potential biologic and molecular differences between epithelial ovarian tumors in different age groups in Chinese cohorts.

## Electronic supplementary material

Below is the link to the electronic supplementary material.

Supplementary Material 1

## Data Availability

The dataset supporting the conclusions of this article is available in the Surveillance, Epidemiology, and End Results (SEER) database. The URL of the database is https://seer.cancer.gov/data. And the raw dataset supporting the results of this study has been included in the supplementary material.
